# Supplementary material for: Study on the effect of modified Liu-an decoction on Th1/Th2 function in Guinea pigs with cough variant asthma through the Notch signal pathway
Source: PLoS One. 2025 Sep 15;20(9):e0327356. doi: 10.1371/journal.pone.0327356 (PMC12435660; doi:10.1371/journal.pone.0327356)
Supplement: Attachment — Submitted filename: REVIEWER REPORT.pdf [file pone.0327356.s001.pdf]

I acknowledge the efforts of the authors in an attempt to provide a therapeutic solution for asthmatic conditions. While the research addresses a clinically relevant question with potentially significant implications for treatment planning, several areas are noted with flaws and clinical concerns. Some of these are summarized below;

MAJOR CONCERNS:

1. **CLINICAL CONCERN:** In this study, the use of numerous herbs (more than 10) appears somewhat arbitrary, suggesting a trial-and-error approach by combining multiple ingredients to achieve results. No identifiable compounds other than concocting too many herbs without analyzing their constituents. This method raises health concerns due to the potential toxicity of unknown constituents. Employing such a mixture without a thorough analysis of each component's active and potentially toxic compounds is neither scientifically rigorous nor acceptable for ensuring safety. Therefore, it is highly important to provide a detailed analysis of the constituents of the herbs used in a study like this, especially for a few key reasons:

a) Pinpoint specific compounds responsible for therapeutic effects to identify the active agents understanding that this knowledge allows researchers to attribute biological activity (such as anti-inflammatory, bronchodilatory, or immune-modulatory effects) to particular compounds. Without this, attributing effects solely to the herbal mixture may limit the scientific rigor of the study and make it difficult to determine which components are beneficial.

b) Detect and manage potentially harmful compounds. Herbs can contain toxic compounds that may pose health risks, particularly if they are administered in higher doses or for prolonged periods. Detailed analysis helps identify any potential toxins, ensuring safer use in clinical or experimental settings.

c) Facilitate consistent replication of treatment effects in future studies. If the herb mixture (like MLAD in this case) is to be recommended for future therapeutic use, having a clear understanding of its components is essential for reproducibility. It ensures that other researchers or clinicians can recreate the mixture with similar composition and dosage, leading to consistent results and therapeutic efficacy.

d) Understand how individual components affect biological pathways. If one herb contains a compound that modulates the Notch signaling pathway (central to the study's hypothesis), identifying it could enhance understanding of how the herbal mixture exerts its effects on Th1/Th2 function.

2. **POOR METHODOLOGY:**

a) Experimental model: Using 3-week-old guinea pigs in a study involving respiratory function, immune response, and airway inflammation seem unethical and thus should be approached with caution. At this age, guinea pigs are quite young, and their respiratory and immune systems are still maturing, which could influence the results and interpretation of the study.

   I. At 3 weeks, guinea pigs are weaned but still undergoing significant physiological development, including in the lungs. Immature lung structure and function could lead to different responses to asthma or inflammation compared to fully mature animals.

   II. The immune response in young animals may not fully mimic that in adults, especially in conditions involving Th1/Th2 balance and chronic inflammation. Young guinea pigs may have an underdeveloped adaptive immune response, which could affect how they respond to allergens and treatments.

III. For studies aimed at understanding asthma-like conditions in humans, using older guinea pigs (e.g., 6–8 weeks or older) would generally provide a more stable model, as their respiratory and immune systems would more closely recapitulating those of adult humans. Young animals might not fully replicate the chronic and complex immune dynamics of asthma, especially cough variant asthma (CVA).

IV. Young animals may experience more stress and potential adverse effects from experimental procedures, particularly if they involve repeated allergen exposure and invasive testing and may therefore violate ethical considerations and animal welfare.

3. Regarding the statement, "*MLAD was composed of Pinellia Ternata 6g, Citrus maxima 6g, Poria Cocos 6g, Prunus Armeniaca 6g, Sinapis Alba 6g, Glycyrrhiza uralensis 3g, pumice 20g, Lepidium Apetalum 6g, Semen Trichosanthis 10g, Rhizoma Arisaema Cum Bile 4g, Raphanus Sativus 10g* ", some concerns are raised;
   I. The statement does not provide any rationale for the specific quantities of each ingredient. While traditional formulations may prescribe certain amounts, discussing why these specific doses are used could enhance understanding, particularly for readers unfamiliar with TCM.
   II. The study failed to show where each of the MLAD composition was sourced; root. fruits, stem or so. Different parts can contain varying concentrations of active compounds and may have distinct therapeutic effects. Including this detail would enhance the study's reproducibility and scientific rigor.
   III. Though minor, while scientific names are provided, they are inconsistent in capitalization and formatting. Scientific names should always be italicized, with the genus capitalized and the species name in lowercase (e.g., *Pinellia ternata*).

4. Under "**1.3 Experimental grouping**", the grouping method used in this study is flawed due to a lack of consideration for body weight balance across the groups. Ensuring that each group has a relatively similar mean body weight is essential for minimizing variability that could affect treatment response and overall outcomes. However, this crucial step was overlooked. In **Table 3**, both the administration of ovalbumin and the treatments appear to be based on body weight when the animals were euthanized for analysis, and for the endpoint outcomes. This oversight introduces a significant bias, as variations in body weight on group level could influence the animals' responses to both the sensitization and treatment, potentially skewing the results. This lack of initial weight-based grouping represents a fundamental methodological flaw that weakens the study's reliability and validity.

5. The **behavioral assessment criteria** used in this study are flawed and inadequate. The criteria for assessing successful model induction—such as nodding, shrugging, and coughing—are subjective and may result in inconsistent evaluations. Implementing a validated scoring model for behavioral assessment, such as the **Asthma Severity Score** or **Pulmonary Symptom Scoring System**, would improve objectivity. Such models assess symptoms based on standardized metrics like cough frequency, respiration rate, and activity level, providing consistent criteria that reduce observer bias and enhance the reproducibility of results.

6. Cyclophosphamide, a powerful immunosuppressant, is administered on Day 1 without clarification of its specific role. Given that CVA is an immune-driven condition, the inclusion

of an immunosuppressant may affect the immune response's development in ways not typical of CVA, potentially influencing the model's relevance.

7. The cytokine analysis in this study is limited by its focus on Th2 responses, limited from Th1.
   a) It omits critical data on Th1 cytokines, such as IFN-γ and other pro-inflammatory markers, which are crucial for understanding the balance between Th1 and Th2 pathways in asthma. The study would benefit from investigating how Th1 cytokines might be downregulated in an asthmatic context, along with potential pathway crosstalk between Notch signaling and Th1/Th2 regulation.
   b) Additionally, the role of effector cells such as macrophages and Tcells (both) in this model remains unexplored. Effector cell activity and differentiation are key to understanding the immune response and would add depth to the study's conclusions. A simple flow analysis could have improved the study rigor.
   c) For robust conclusions on cytokine expression, it's essential to account for possible confounding factors such as underlying health conditions or infections (e.g., intestinal or blood pathogens) that can influence cytokine levels. Cytokine regulation is heavily influenced by the JAK-STAT pathway; yet, the study lacks details on how, or if, JAK-STAT interacts with Notch signaling. Clarifying these pathway interactions would strengthen the study's insights into the mechanisms at play in asthma.

8. The authors claimed that they stained Masson Trichrome for fibrosis analysis in the lungs, only to be seen on the section slide. In fact, I had expected IHC stain to establish the findings here but to no avail.

MINOR CONCERNS:

1. The "Conclusion" section is placed before the "Discussion." This is unconventional; the authors should justify this arrangement or consider reordering the sections.

2. There are numerous capitalization errors throughout the manuscript that need to be addressed.

3. In the dosing description, there's an error in consistency and clarity: "*Low-dose MLAD decoction group: oral administration of 0.74g/mL MLAD solution, 1mL/100g/d, once a day. Mid-dose MLAD group: oral administration of 1.48g/mL MLAD solution, 1mL/100g/d, once a day. <u>Low-dose</u> MLAD group: oral administration of 2.22g/mL MLAD solution, 1mL/100g/d, once a day. The three concentration groups of MLAD are equivalent to 1:2:3 of the adult dosage*." Something is wrong here. This should be clarified and corrected to accurately describe each dosage group.

4. The statement "*From the day of stimulation, that is, from the 23rd day of the experiment, 30 minutes before the stimulation with a nebulizer, each group was given intragastric administration intervention for 14 days*" lacks specificity. What was the purpose of this intervention?

5. The sentence, "*The dosage for each group was calculated according to the conversion formula of body surface area between humans and guinea pigs*," is vague and should be clarified, perhaps with the formula provided for transparency.

6. Avoid starting sentences with numerals, as in "*60 healthy 3-week-old pure male guinea pigs...*" Instead, write, "*Sixty (60) healthy 3-week-old male guinea pigs...*" This applies throughout the manuscript.

7. Some sentences are overly lengthy and difficult to follow, such as: "*Through our previous animal experimental studies, we discovered that MLAD could reduce the levels of serum tumor necrosis factor-α (TNF-α) and interleukin-5 (IL-5) in CVA rats, reduce the number of eosinophils (EOS) in the bronchoalveolar lavage fluid (BALF), reduce airway inflammation and airway collagen deposition, thereby improving the pathological state of airway remodeling and reducing cough frequency*." This should be broken down for clarity.

8. The authors mention a previous clinical trial: "*Our previous clinical trials have shown that treatment of CVA using Modified Liu-an Decoction (MLAD) and Montelukast sodium was effective in children with phlegm-heat and phlegm-dampness syndromes*." Specific details, including location and timing, should be provided to give context to this claim.

9. Many statements appear to be derived from the literature without proper citation. For instance, "*The etiology and mechanism of CVA are complicated, and antibiotic treatment is ineffective,*" lacks a reference.

10. The abbreviation "CVA" is used without being defined at first use. It should be spelled out initially for clarity.

11. There is no "Introduction" section, which is unusual for a scientific manuscript.

12. The introduction should provide a rationale, emphasizing how this new agent could potentially offer advantages over current treatments and why it is important to pursue this line of research.

13. The poor design and readability of figures and graphs diminish the accessibility and interpretability of the data. Enhanced visuals with clear labeling would allow readers to better understand the results.

In summary, the study would benefit from a stronger structural foundation, improved clarity and consistency, more rigorous methodology, comprehensive data analysis, detailed justification for MLAD's use, and enhanced figures. Addressing these points would lead to a more reliable and scientifically robust study.
