# Supplementary material for: Study on the effect of modified Liu-an decoction on Th1/Th2 function in Guinea pigs with cough variant asthma through the Notch signal pathway
Source: PLoS One. 2025 Sep 15;20(9):e0327356. doi: 10.1371/journal.pone.0327356 (PMC12435660; doi:10.1371/journal.pone.0327356)
Supplement: Attachment — Submitted filename: The Reply to reviewers and the Editor.docx [file pone.0327356.s004.docx]

**The Reply to reviewers and the Editor**

**Title: Study on the Effect of Modified Liu-an Decoction on Th1/Th2 Function in Guinea Pigs with Cough Variant Asthma through the Notch Signal Pathway**

**Author information**

Fangwei Xu ^1^ Jian Deng^2^ Ningning Zhang^3^ LEOW XinTing Kimberly^4^ Panpan Li^5^ Chen Lu^6^ Yuhang Chen^7^ Ye Zhang^8^ Liqun Wu^9*^

Respondence to Liqun Wu*

**Reply to Reviewer 3,**

Thank you for your valuable guidance and comments.

**Major revisions：**

Q1: There are some format errors. For example, in the first line of the second paragraph of 2.1 General condition of guinea pigs in each group, the 7th should be 7th ("th" superscripted). The spacing before and after punctuation marks (such as “,”, “<” and “±”) is inconsistent. Also, the "n"s indicating sample size and the "P"s indicating significance should be italicized. The capitalization of 3.3 is not consistent with that of 3.1 and 3.2. I hope the authors can improve the format of this manuscript.

Authors：Formatting issues have been corrected
Q2: Figure 1, 3, 4, 6, 7, 8 should each include a title, not just a legend.

Authors：The title of the tables have been added
Q3: In Figure 7, the protein weights should be written as kDa and the font should be standardized (I suggest that they be standardized to Arial).

Authors：Formatting issues have been corrected
Q4: I suggest that the bars in Figure 8 should be grouped by proteins, rather than experimental groups. This could help with the clarity of the results. Also, I think the * and # showing significance are misplaced, as they are not positioned directly above the bars.

Authors：Experimental groupings were used because it was felt that they needed to be viewed in conjunction with Figure 7.The *'s and #'s have been relabeled in the appropriate places.
Q5: Is it possible to add some indicators that can directly demonstrate Th1/Th2 conversion, such as ① flow cytometry or ② the detection of Th1 marker T-box transcription factor (T-bet) and Th2 marker GATA-binding protein 3 (GATA-3) or ③ Th1-associated proinflammatory cytokines interferon (IFN)-γ and tumor necrosis factor (TNF)-α by CD4+ T cells?

Authors：We sincerely thank the reviewers and editors for their comments and suggestions. We agree that further studies will illustrate the effect of this experiment on Th1/Th2 balance using flow cytometry assays, and that tumor necrosis factor (TNF)-α has been demonstrated to be essential for its specific role in cough-variant asthma in previous experimental studies. However, due to the current limitations of our research team, including laboratory infrastructure, funding, and personnel, we were unable to conduct an in-depth study at this stage.

We hope that this manuscript will serve as a foundational study that will support future research grant applications and enable us to conduct further experiments. In future studies, we will consider including the detection of indicators such as the Th1 marker T-box transcription factor (T-bet) and the Th2 marker GATA-binding protein 3 (GATA-3) to further validate the specific mechanism of the Th1/Th2 balance both in vivo and in an in vitro model of cough-variant asthma. In addition, we would like to explore the exact mechanism of airway mucus hypersecretion in cough-variant asthma.

Q6: I suggest that the original Western Blot data should be provided in supplementary materials.

Authors：Raw data have been added to the annex.
Q7: There are some Chinese characters in the 3 Discussion part of Revised Manuscript with Track Changes.

Authors：The Chinese characters are actually translations of the corresponding part, which has been deleted

**Reply to Reviewer 4,**

Thank you for your valuable guidance and comments.

Comments:
1.The study design includes six groups labeled A-F, where Group A is the blank control group, Group B is the model group, and the remaining groups (C-F) can be referred to as model drug groups (MS group and MLAD groups). I believe the primary role of Group A is to validate the successful modeling of Groups B-F. The authors also indicate that the animal model is mainly evaluated based on behavioral performance, with the effective observation indicators being 1.6.1-1.6.2. When analyzing the experimental data, considering the influence of a single factor, comparisons between the blank control group and the model group, as well as between the model group and the model drug groups, are statistically meaningful. However, in the description of the experimental results, there are comparisons between the blank control group data and the model drug group data, such as in 2.1 regarding diet and weight, 2.2 regarding airway sensitivity, 2.3 regarding airway hyperresponsiveness, and 2.6 regarding lung tissue of guinea pigs in each group.
Authors：An experimental study reported that successful modeling was demonstrated when guinea pigs coughed more than 5 times after capsaicin stimulation with nodding of the head, shrugging of the fur, rubbing of the face, irritability, or sluggishness.So all were successful in modeling. And the final experimental results of the model group and the medicated group were compared because the node of comparison was after the drug intervention
2.The manuscript exhibits a lack of precision in its presentation. For instance, in the Introduction, the phrase "[7]" is used; in Section 1.2, the notation "AI(OH)3" appears; in Section 1.3, the text states "divided into 5 groups"; in Section 1.5, there is a duplication of "E group" and an omission of "F group"; in Section 1.7, the term "mean±standard" is employed; and in Sections 2.4 and 2.7, the expression "the normal group" is utilized. These inaccuracies and inconsistencies require correction.
Authors：Formatting issues have been corrected
3.Certain paragraphs are overly lengthy and would benefit from appropriate segmentation. For example, in the Discussion, the second paragraph regarding Airway Inflammatory, as well as the sections on Airway Hyperresponsiveness and airway remodeling, could be restructured for improved clarity and readability.
Authors：Some have been appropriately scaled down
4.In the Introduction, the authors state, "Through our previous animal experimental studies, we discovered that MLAD could reduce the levels of serum tumor necrosis factor-α (TNF-α) and interleukin-5 (IL-5) in CVA rats, reduce the number of eosinophils (EOS) in the bronchoalveolar lavage fluid (BALF), reduce airway inflammation and airway collagen deposition, thereby improving the pathological state of airway remodeling and reducing cough frequency." However, in the Results section of the Abstract, the authors mention, "Compared with the model group, MLAD reduced the inflammatory infiltration and collagen deposition in the lungs of CVA guinea pigs (P<0.05)." This raises the question of whether the same data has been reported in multiple publications.

Authors：The previous experiment was done on rats and this experiment is on guinea pigs, for different data.The previous study was based on the CVA rat model, which mainly focuses on the role of MLAD in the regulation of Th2-type inflammatory factors (TNF-α, IL-5) and airway EOS infiltration. In contrast, the current study used the CVA guinea pig model, which is a preferred model for the study of chronic cough and airway hyperresponsiveness because the airway innervation and cough reflex sensitivity of guinea pigs are closer to those of humans. Although “lung inflammatory infiltration” and “collagen deposition” are common indicators for assessing airway remodeling, all the data from the guinea pig experiments in this study are reported for the first time. We validated the cross-species efficacy of MLAD in a new animal model and further revealed its generalized effect on airway remodeling, which provides a more comprehensive chain of evidence for subsequent clinical translation. While the reference to rat experiments in the introduction was intended to illustrate the basis of the previous work, the focus on the guinea pig model in the present study was intended to follow up on the effect of adding MLAD on the modulation of neurogenic airway inflammation and to further explore its effect on airway inflammation-remodeling coupling in a guinea pig model with enhanced neurosensitivity

5.Due to the current font used, certain details in the figures are difficult to discern, particularly in Fig. 2, Fig. 3, and Fig. 4. It is recommended to enhance the clarity and resolution of these figures to ensure accurate interpretation of the data.

Authors：We sincerely appreciate your constructive feedback. We have thoroughly reviewed the manuscript and implemented corresponding revisions, including modifications to relevant figure legends for improved clarity. Figure 2 was exported from another machine, so there is no way to change the clarity, Figure 3 Figure 4 has been modified
